# Role of zinc in health and disease

**DOI:** 10.1007/s10238-024-01302-6

**Published:** 2024-02-17

**Authors:** Lucy I. Stiles, Kevin Ferrao, Kosha J. Mehta

**Affiliations:** 1 Faculty of Life Sciences and Medicine, GKT School of Medical Education, King’s College London, London, UK; 2 Faculty of Life Sciences and Medicine, Centre for Education, King’s College London, London, UK

**Keywords:** Zinc, ZIP, ZnT, Acrodermatitis enteropathica, Zinc deficiency, Zinc toxicity

## Abstract

This review provides a concise overview of the cellular and clinical aspects of the role of zinc, an essential micronutrient, in human physiology and discusses zinc-related pathological states. Zinc cannot be stored in significant amounts, so regular dietary intake is essential. ZIP4 and/or ZnT5B transport dietary zinc ions from the duodenum into the enterocyte, ZnT1 transports zinc ions from the enterocyte into the circulation, and ZnT5B (bidirectional zinc transporter) facilitates endogenous zinc secretion into the intestinal lumen. Putative promoters of zinc absorption that increase its bioavailability include amino acids released from protein digestion and citrate, whereas dietary phytates, casein and calcium can reduce zinc bioavailability. In circulation, 70% of zinc is bound to albumin, and the majority in the body is found in skeletal muscle and bone. Zinc excretion is via faeces (predominantly), urine, sweat, menstrual flow and semen. Excessive zinc intake can inhibit the absorption of copper and iron, leading to copper deficiency and anaemia, respectively. Zinc toxicity can adversely affect the lipid profile and immune system, and its treatment depends on the mode of zinc acquisition. Acquired zinc deficiency usually presents later in life alongside risk factors like malabsorption syndromes, but medications like diuretics and angiotensin-receptor blockers can also cause zinc deficiency. Inherited zinc deficiency condition acrodermatitis enteropathica, which occurs due to mutation in the *SLC39A4* gene (encoding ZIP4), presents from birth. Treatment involves zinc supplementation via zinc gluconate, zinc sulphate or zinc chloride. Notably, oral zinc supplementation may decrease the absorption of drugs like ciprofloxacin, doxycycline and risedronate.

## Introduction

### Significance of zinc in human health

Zinc cannot be synthesised within the human body, so external intake of zinc is essential to maintain adequate levels in the body [1]. It is the second most abundant trace element in the body, after iron [2]. One in ten proteins found in the body is a zinc protein [3], and more than 300 enzymes and 1000 transcription factors depend on zinc for their activities [4]. Thus, zinc is an essential micronutrient involved in many cellular processes such as protein synthesis, nucleic acid metabolism including DNA synthesis, gene transcription [1], cell proliferation and differentiation, and mitosis [5].

These zinc-requiring cellular processes extend the significance of zinc to physiological level. For example, zinc is a structural component of the bone tissue and plays a role in collagen matrix synthesis, mineralisation, and bone turnover [6]. Also, zinc regulates intracellular signalling pathways of innate and adaptive immune cells [7], influences immune responses including antibody production, inflammatory signalling and lymphocyte differentiation [8], and thereby plays an essential role in the functionality of the immune system.

Zinc also plays a role in the endocrine system. For example, zinc is required in the formation and structural stability of insulin [9]. Essentially, insulin dimers form hexameric units, coordinated by two zinc ions in the central axis of the hexamer [10]. No wonder the beta cells of the pancreas contain significantly higher concentrations of zinc than other cells of the body. Furthermore, zinc ions act on the insulin signalling pathway and stimulate lipogenesis and glucose uptake into the adipocytes [9]. Zinc transporter (ZnT)-8 mediates signalling between the pancreas and liver to allow optimal insulin release, while zinc/iron-regulated-transporter-like-protein-(ZIP)7 is thought to play a role in glycaemic control within skeletal muscle. [9].

Thyroid hormones are involved in many physiological functions, such as the anabolism of proteins and increasing the basal metabolic rate and bone growth in children [11]. Zinc plays an important role in the metabolism of thyroid hormones. It regulates the synthesis of thyroid-releasing hormone (TRH) and thyroid-stimulating hormone (TSH). Zinc modulates their structure and thereby regulates the transcription factors which are essential for thyroid hormone synthesis [12]. Thus, in humans with zinc deficiency, levels of TSH, serum triiodothyronine (T3) and thyroxine (T4) also decrease [13], with several studies suggesting zinc deficiency as a cause of subclinical hypothyroidism [14]. Unsurprisingly, zinc supplementation appears to enhance thyroid hormone levels, particularly T3 [15].

Zinc is essential for male fertility. A zinc-sensing receptor, known as GPR39, has been found in the sperm tail and acrosome. When extracellular Zinc(II) binds to this receptor, it triggers an intracellular signalling pathway that ultimately results in increased sperm motility and acrosomal exocytosis [16]. Thus, zinc could have a role in the prevention, diagnosis and treatment of male infertility [17].

Additionally, zinc is important for the normal development and functioning of the central nervous system (CNS). Zinc balance is vital for neural tube formation and stem cell proliferation during development. Various zinc-dependent enzymes contribute to the function of the CNS, and ‘free’ zinc appears to modulate a variety of post-synaptic receptors. For example, zinc inhibits GABA-A receptors, which reduces their inhibitory actions. Alterations in zinc levels thereby affect the CNS and play a role in conditions such as Alzheimer’s disease and depression [18].

The human retina contains zinc in high concentrations [19]. Studies have suggested a link between higher anti-oxidant intake, including zinc (likely because zinc is a cofactor of superoxide dismutase, an anti-oxidant enzyme and also because zinc is an inhibitor of NADPH oxidase [20], which catalyses the production of reactive oxygen species [21]) and a decreased risk of age-related macular degeneration (AMD) [22], a leading cause of vision loss [23]. Studies have proposed the benefits of anti-oxidant supplementation, including zinc, in slowing the progression of AMD through the prevention of cellular damage in the retina [24].

Importantly, randomised trials in children six months to twelve years of age showed a positive effect of zinc supplementation in reducing all-cause and infectious disease mortality. It also showed a minor positive impact on linear growth [25].

Collectively, these examples highlight the significance of zinc in human health.

### Micro-deficiencies and prevalence of zinc deficiency

It is estimated that 372 million (56%) preschool-aged children and 1.2 billion (69%) non-pregnant women of reproductive age across the globe have a deficiency in at least one of the micronutrients, namely zinc, folate, vitamin A and iron. Geographically, 75% of micronutrient-deficient preschool-aged children live in South Asia, sub-Saharan Africa, or East Asia and the Pacific. 57% of micronutrient-deficient non-pregnant women of reproductive age live in East Asia and the Pacific or South Asia [26]. An estimated 17% of the world’s population is at risk of insufficient zinc intake [1, 27]. In Southeast Asia and sub-Saharan Africa, zinc deficiency is endemic, affecting up to 33% of the population. Zinc deficiency is also prevalent in Turkey, Egypt, and Iran due to high phytate intake in their diets, which decreases zinc absorption and, therefore, its bioavailability. Other countries have a markedly lower prevalence of zinc deficiency, notably China, where its incidence decreased from 17 to 8%, as recorded in 2005 [1].

## Zinc uptake, absorption, and regulators of its bioavailability

### Zinc: location, dietary sources, and intake recommendation

Table 1 provides an overview of the level and proportion of zinc at physiological and cellular levels in the human body.

**Table 1:** Zinc levels in a healthy human

Compartments	Levels of zinc
Serum (Normally holds < 1% of total body zinc) [28, 29]	70–250 µg/dL [1] 109–130 µg/dL [30] *62.13–117.72 µg/dL (conversion based on 9.5–18 µM [31]) *78.48 µg/dL–104.64 µg/dL (conversion based on 12–6 µM [29]) 60–120 µg/dL, (59–125 μg/dL for male and 50–103 μg/dL for female) in Bangladesh sample population [32]
Tissues	Muscles store about 50 to 60% of the zinc found in the body [3, 29], followed by bones which have about 30 to 36.7% [3, 29], then skin (4.2%) and liver (3.4%) [29] Prostate, pancreas, and bone, have high zinc concentration ranging from 100 to 250 µg/g [33] Heart, brain, and plasma, have comparatively lower concentration, ranging from 1 to 23 µg/g [33]
Intracellular distribution	30–40% in nucleus, 50% in cytoplasm, and remaining 10–20% associated with membrane organelles [34, 35] Mitochondria (0.14 pM), the mitochondrial matrix (0.2 pM), the endoplasmic reticulum (0.9 pM-5 nM), and the Golgi apparatus (0.2 pM) [28]
Total levels in an adult body	2–3 g [28, 36]

*Conversion performed using MediCalc available at https://www.scymed.com/en/smnxtb/tbcbpgh1.htm

Zinc cannot be stored in substantial amounts, and so, regular dietary intake is essential to ensure sufficient zinc availability [17]. Dietary sources of zinc include fish, oysters, meat, legumes, nuts, beans, whole grains, beef, eggs, and dairy. Oysters are the richest source of zinc, while fruits and vegetables are the poorest source. Although beans, nuts, and whole grains contain zinc, the bioavailability of zinc from these is lower than food from animal sources due to the presence of phytates [37].

There are differences in the dietary recommendations of zinc. Data around this include recommendations of 7.4 mg/day (approximately) [38]. In the US, recommendations include 15 mg/day [30], 11 mg/day [39] and 11 mg/day and 8 mg/day for adult men and women, respectively, who are age 19 and above [37]. In the UK, the recommendation is 9.5 mg/day for an adult man and 7 mg/day for an adult woman. The UK Department of Health recommends that zinc intake should not exceed 25 mg/day [40].

### Zinc transporters: ZIPs and ZnTs

During digestion, zinc is released from food as free Zn^2+^ ions. These ions need to be transported from the intestinal lumen to the enterocyte, then from here into the circulation and from circulation to the cells that require zinc for their activities. Also, within cells, there is intracellular zinc movement and compartmentalisation. These zinc transport processes are facilitated by two important types of zinc transporters: Zinc/iron-regulated-transporter-like-proteins (ZIPs) and Zinc transporters (ZnTs). ZIPs increase intracellular/cytoplasmic zinc levels by transporting zinc from the extracellular space and/or intracellular organelles into the cytoplasm. In general, ZnTs (the exception is ZnT5B, which is a bidirectional transporter) reduce intracellular/cytoplasmic zinc by transporting zinc from the cytoplasm to extracellular space (promoting zinc efflux from cells) or into an organelle for its compartmentalisation [38]. ZnTs and ZIPs are located in several different tissues/cells, on different regions of the cell surface, and on the surface of intracellular organelles (Tables 2 and 3).

**Table 2:** ZnT transporters: location and regulation

Transporter	Tissue and cellular distribution	Stimulus	Response	Mechanism of response
ZnT1	Ubiquitous [41], with notable abundance in the duodenum, jejunum [42] and kidney [43] Plasma membrane (basolateral region in epithelial cells and apical region in pancreatic acinar cells) and vesicles [43, 44]	Increased cellular zinc in HepG2 cells [44, 45]	Increase in ZnT1 mRNA [44, 45]	Metal-response element-binding transcription factor-1 (MTF-1) binds to metal-response elements (MREs) in ZnT1 promoter [45]
		Zinc deficiency in HepG2 cells [45]	Decreased ZnT1 protein in HepG2 cells [45]	Endocytosis of cell surface ZnT1 with subsequent degradation via proteasomal or lysosomal pathways [45]
		Lipopolysaccharide in dendritic cells [44, 46]	Increase in ZnT1 mRNA [44, 46]	Process mediated via Toll/interleukin-1 receptor (TRIF) in Toll-like receptor (TLR) signalling [44, 46]
		T-cell stimulation by phytohaemagglutinin (immune activation) [47]	Downregulation of ZnT1 mRNA expression in T-cells [47]	–
ZnT2	Vesicles, secretory granules [43] Retina, mammary glands, small intestine, pancreas, kidney, prostate [44] Two variants: One variant is primarily located on the membranes of vesicles, including endosomes and lysosomes [44, 48] as well as zymogen granules in pancreatic acinar cells and the inner mitochondrial membrane in mammary cells [44] The other variant is localised to the plasma membrane [44, 48]	High zinc levels in mammary glands, prostate, pancreas, small intestine, kidney, and retina [44]	Upregulation of ZnT2 mRNA [44]	MTF-1 binding to MRE downstream from ZnT2 transcription start site [44, 49]
		Glucocorticoid hormone in pancreatic acinar cells [44]	Upregulation of ZnT2 transcription [44]	Glucocorticoid receptor and STAT5 interaction [44, 49]
		Prolactin in mammary epithelial cells [44]	Upregulation of ZnT2 transcription [50]	Prolactin induced JAK2/STAT5 signalling pathway [50]
			Decreased ZnT2 expression [44, 51]	Prolactin induced post-translational ZnT2 ubiquitination [44, 51]
ZnT3	Protein detected in brain, retina, and pancreas. RNA detected in testis, duodenum, airways and adipose tissue [52] On the membranes of synaptic vesicles [44, 53]	Angiotensin II in vascular smooth muscle cells [44, 54]	Downregulation of ZnT3 mRNA expression [54]	Angiotensin II leads to reactive oxidative species which is thought to downregulate ZnT3 [54]
ZnT4	Ubiquitous, with greater abundance in the brain and digestive tract [44] Trans-golgi network, cytoplasmic vesicles, endosomes, lysosomes, and Golgi apparatus [44]	Increased extracellular zinc [43]	Expression may not be affected but ZnT4 trafficking is induced [43]	Trafficking occurs from trans-golgi network to cytoplasmic vesicular compartment in cultured NRK cells [43]
		T-cell stimulation by phytohaemagglutinin (immune activation) [47]	Downregulation of ZnT4 mRNA expression in T-cells [47]	–
		Lipopolysaccharide in dendritic cells [46]	Upregulated expression of ZnT4 mRNA transcripts [46]	This is mediated via Toll/interleukin-1 receptor (TRIF) and myeloid differentiation primary response 88 (MyD88) protein in Toll like receptor (TLR) signalling [46]
		Granulocyte–macrophage colony-stimulating factor in macrophages [44]	Upregulation of ZnT4 mRNA expression [44]	–
		Cell differentiation in villus of small intestine [43, 44]	Increased ZnT4 expression [44]	–
ZnT5	ZnT5 mRNA was found in human endocrine pancreas, prostate and testis [55]. Also found in small intestine [56] Two variants: Variant A is located at the Golgi apparatus [57] Variant B is a bidirectional transporter located throughout the cell, including at the plasma membrane [44, 57] and is on the apical surface of enterocytes [58]	High or low zinc levels [43]	Increased expression [43] Decreased expression [43]	Increased mRNA stability [43] Transcriptional repression [43], which is under control of the zinc transcriptional regulatory element (ZTRE) [44]
		Lipopolysaccharide in mice liver [59]	Increased ZnT5 mRNA [59]	–
ZnT6	Protein detected in mouse brain, lung, small intestine, and kidney [60] Trans-golgi network, Golgi apparatus [44, 60]	T-cell stimulation by phytohaemagglutinin (immune activation) [47]	Downregulation of ZnT6 mRNA expression in T-cells [47]	–
		Lipopolysaccharide in dendritic cells [46]	Upregulation in ZnT6 mRNA expression [46]	Mediated through the Toll/interleukin-1 receptor (TRIF) and myeloid differentiation primary response 88 (MyD88) protein in Toll-like receptor (TLR) signalling [46]
ZnT7	In mice, protein was found in lung and small intestine. The mRNA was found in liver, kidney, spleen, heart, brain, small intestine, and lung, with abundant expression in small intestine and liver and less expression in heart [61] Early secretory pathway including Golgi apparatus [44]	T-cell stimulation by phytohaemagglutinin (immune activation) [47]	Downregulation of ZnT7 mRNA expression in T-cells [47]	–
		Granulocyte–macrophage colony-stimulating factor in macrophages [44]	Upregulation of ZnT7 mRNA expression [44]	–
ZnT8	Pancreas [62]; pancreatic *β*-cell-specific zinc transporter [44] on the membranes of insulin secretion granules [63]	Acute exposure to cytokines (including IL-1*β*, IFN-*γ*, IL-17, TNF*α*) in EndoC-*β*H1 cells [64]	Downregulation of ZnT8 protein [64]	–
ZnT10	Liver, brain [62] and intestine [65] Early/recycling endosomes, Golgi apparatus but can localise to plasma membrane under high extracellular zinc concentrations [44]	IL-6 in human SH-SY5Y neuroblastoma cells [44]	Decrease in both ZnT10 mRNA and protein levels [66]	IL-6 may affect the transcription of the *SCL30A10* (gene encoding ZnT10), possibly involving a regulation element [66] which is suggested to be the ZTRE [44]
		Angiotensin II in vascular smooth muscle cells [44, 54]	Downregulation of ZnT10 mRNA expression [54]	Angiotensin II leads to reactive oxidative species which is thought to downregulate ZnT10 [54]
		High manganese intake in mice [67]	Increased ZnT10 protein levels in liver and small intestine in male mice [67]	–
		High extracellular zinc levels in human 5Y5Y neuroblastoma cells [68]	Downregulation of ZnT10 mRNA [68]	A zinc responsive element (ZRE) may be involved in ZnT10 downregulation [68]

**Table 3:** ZIP transporters: location and regulation

Transporter	Tissue and cellular distribution	Stimulus	Response	Putative mechanism of response
ZIP1	Ubiquitous, [69] Plasma membrane [44] Intracellular vesicles [69]	Zinc deficiency in vitro [70]	Increased mouse ZIP1 protein expression in transfected Human embryonic kidney cells (HEK293) [70] (ZIP1 expression was unaffected by zinc in vivo [71])	Reduced rates of ZIP1 endocytosis due to zinc limitation [70]. Endocytosis of ZIP1 mediated through a di-leucine sorting signal [72]
		Cell differentiation of pluripotent mesenchymal stem cells into osteoblast-like cells [73]	Increased ZIP1 protein expression [73]	–
ZIP2	Dendritic cells, ovaries, skin, liver [79] Plasma membrane [79]	Reduced intracellular zinc in monocytes [44, 74]	Upregulation of ZIP2 mRNA in monocytes [44, 74]	–
		Granulocyte macrophage-colony stimulating factor in macrophages [44]	Upregulation of ZIP2 mRNA in macrophages [44]	–
		Keratinocyte differentiation [44]	Upregulation of ZIP2 mRNA in differentiating keratinocytes [44]	–
		Macrophage polarisation to M2 [75]	Increased ZIP2 mRNA levels [75]	–
ZIP3	Widespread [69] Plasma membrane but can localise to intracellular compartments after zinc treatment [44]	Zinc deficiency in zebrafish gill [76]	Increased ZIP3 mRNA [76]	–
		Zinc deficiency in vitro [70]	Increased cell surface mouse ZIP3 expression in transfected cells [70]	Reduced rates of ZIP3 endocytosis due to zinc limitation [70]
		Prolactin in secretory mammary epithelial cells [77]	Upregulation of ZIP3 mRNA and protein levels [77]	–
ZIP4	Small intestine and epidermis [79] Plasma membrane [79]	Cytosolic zinc excess [28, 44]	Downregulation of ZIP4 protein [44]	Endocytosis and degradation ubiquitin-proteasomal and lysosomal degradation pathways [44] Zinc repletion can lead to endocytosis and degradation of ZIP4 and ZIP4 mRNA destabilisation [71]
		Zinc deficiency [28, 44]	Upregulation of ZIP4 [28, 44]	Non-transcriptional: ZIP4 mRNA stabilisation [44] Transcriptional: Transcriptional upregulation mediated by Krüppel-like factor 4 (KLF4) [43, 78] Post-translational modification: Proteolytic cleavage of extracellular amino-terminal ectodomain [28, 43, 44]
ZIP5	Intestine, kidney, liver and pancreas [69, 79] Plasma membrane [43, 79]	Zinc availability in mice [44, 80]	Upregulation of ZIP5 translation [44, 80]	Facilitated by a conserved stem-loop and two overlapping miRNA seed sites in the 3’-untranslated region [44, 80]
		Dietary zinc deficiency in mice [71]	Downregulation of ZIP5 translation [71]	ZIP5 mRNA is associated with polysomes and ZIP5 protein is endocytosed and degraded in enterocytes, acinar cells, and endoderm cells [71]
ZIP6	Widespread [69, 79] Plasma membrane [44]	Lipopolysaccharide in dendritic cells [46]	Downregulation of ZIP6 mRNA expression [46]	Mediated through Toll/interleukin-1 receptor (TRIF) in Toll like receptor (TLR) signalling [46]
		Lipopolysaccharide in mice liver [59]	Increased ZIP6 mRNA [59]	–
		Macrophage polarisation to M2 [75]	Increased ZIP6 mRNA [75]	–
ZIP7	Widespread [69, 79]. Colon, skin [79] Endoplasmic reticulum and golgi apparatus [44]	Supplemental zinc [43]	Protein abundance of ZIP7 repressed by supplemental zinc [43]	–
		Cellular zinc levels [81]	ZIP7 expression inversely correlate with cellular zinc levels in CLN6 neurons [81]	
		Macrophage polarisation to M2 [75]	Increased ZIP7 mRNA levels [75]	–
ZIP8	Widespread [69, 79, 82], T-cells [69], highest levels in the lung [82] Plasma membrane (apical in polarised cells) and lysosome [44]	T-cell activation in vitro [83]	Upregulation of ZIP8 expression in human T-cells [83]	–
		Lipopolysaccharide in primary human lung epithelia, monocytes and macrophages [84]	Upregulation of ZIP8 at transcriptional level [84]	NF-κB-dependent mechanism [84]
		TNF-alpha in primary human lung epithelia, monocytes and macrophages [84]	Upregulation of ZIP8 at transcriptional level [84]	NF-κB-dependent mechanism [84]
		Iron loading in rat H4IIE hepatoma cells [85]	Increase in total and cell surface ZIP8 levels [85]	–
ZIP9	Widely distributed [79] Plasma membrane, golgi apparatus [44]	Macrophage polarisation to M2 [75]	Increased ZIP9 mRNA levels [75]	–
ZIP10	Brain, liver, erythroid cell, kidney [69], renal cell, carcinoma B cell [79] Plasma membrane [43]	Zinc deficiency in zebrafish gill [76] Zinc excess in vitro and in vivo [76]	Upregulation of ZIP10 mRNA [76] Downregulation of ZIP10 mRNA [76]	MTF-1 was suggested to be a negative regulator of ZIP10 expression [76]
		Zinc deficiency in mice brain and liver [86]	Upregulation of ZIP10 transcription [86]	During zinc sufficient conditions, zinc-activated MTF-1 physically blocks Pol II movement through the gene, leading to ZIP10 transcription downregulation [86]
		Lipopolysaccharide in dendritic cells [46]	Downregulation of ZIP10 mRNA transcript expression [46]	Mediated through Toll/interleukin-1 receptor (TRIF) in Toll-like receptor (TLR) signalling [46]
		Cytokines in early B cell developmental stages [87]	Upregulated ZIP10 transcription [87]	JAK/STAT pathway involving two STAT binding sites in the promoter [87]
		Thyroid hormone in intestine and kidney cells in a rat model of hypo- and hyperthyroidism [88]	Increased ZIP10 mRNA and protein levels in hyperthyroid rats and decreased ZIP10 mRNA in hypothyroid rats, when compared to euthyroid rats [88]	–
ZIP11	Suggested to localise to stomach and colon [82] Nucleus, intracellular vesicles and plasma membrane of stomach and colon, golgi in mammary epithelial cells [44, 82]	Possibly zinc-dependent [89]	ZIP11 expression only modestly decreased in mouse stomach but not large or small intestine in response to dietary zinc deficiency. Upon acute zinc repletion, expression levels were not restored [89]	The presence of many MREs upstream of the first exon of the ZIP11 gene would suggest that ZIP11 expression is upregulated in response to increasing zinc levels; however, this was not seen in practice [44]
ZIP12	Brain [69, 79, 82], testis and retina [69], pulmonary vascular smooth muscle [79] Plasma membrane [44]	Hypoxia in pulmonary vascular smooth muscle cells [90]	Upregulation of ZIP12 mRNA expression [90]	The *Slc39a12* gene contains a hypoxia response element (HRE) encoding HIF-1*α*- and HIF-2*α*-binding motifs and is located 1 kb downstream of the ZIP12 transcription start site [90]
ZIP13	Widespread [69], hard and connective tissues [79], golgi apparatus, and cytoplasmic vesicles [44]	High iron levels in Drosophila [91]	Upregulation of Drosophila ZIP13 levels [91]	Iron stabilises Drosophila ZIP13 protein, protecting it from degradation [91]
ZIP14	Widespread, liver, bone, and cartilage [79] Plasma membrane [44], endosome [79]	Zinc deficiency in mouse liver [92]	Upregulation of ZIP14 expression [92]	Mediated through the UPR [92]
		IL-6 in mouse hepatocytes [59]	Increased ZIP14 mRNA and protein [59]	–
		Inflammation induced by turpentine [59]	Increased ZIP14 mRNA [59]	Requires IL-6 [59, 93]
		Lipopolysaccharide in mice liver [59]	Increased ZIP14 mRNA [59]	Partially requires IL-6 [59, 93]
		Nitric oxide (induced by IL-1*β*) in mice liver [93]	Increased ZIP14 transcription [93]	Nitric oxide increases binding of Activator Protein-1 (AP-1) to the ZIP14 promoter [93]
		High manganese intake in mice [67]	Upregulated liver ZIP14 expression in both male and female mice, but upregulated small intestine ZIP14 expression only in male mice [67]	–
		High extracellular glucose (medium) involving INS-1E cells [94]	Upregulation of ZIP14 mRNA expression [94]	–
		Iron loading in rat liver and pancreas, and in hypotransferrinemic mice liver [95]	Upregulated ZIP14 protein expression [95]	–

### Process of zinc uptake, absorption, and circulation

**Fig. 1:**
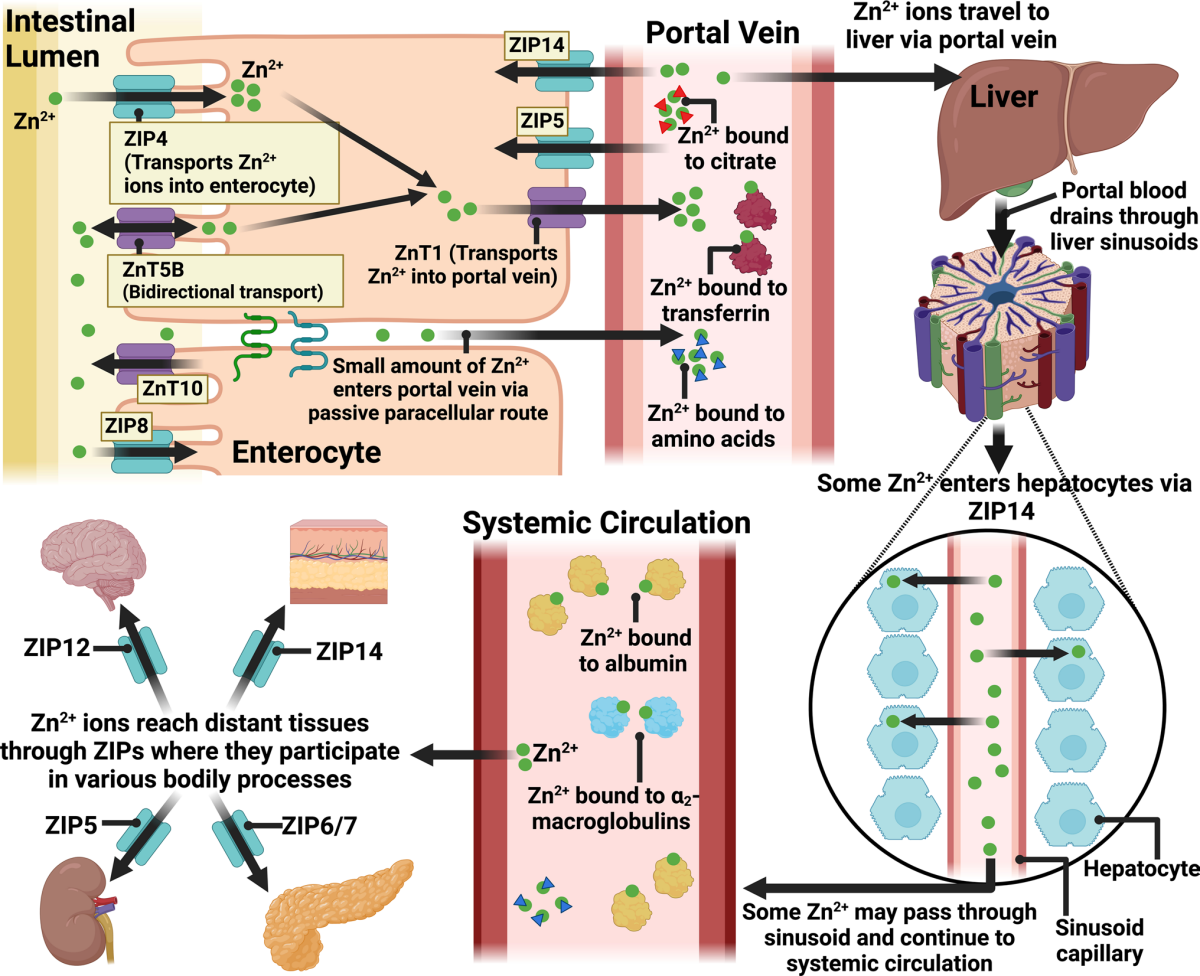
Zinc uptake under physiological conditions. Zinc ions are transported from the intestinal lumen into the enterocyte by ZIP4. Other zinc transporters on the apical membrane include ZIP8 [101], ZnT10 [67] and ZnT5B [56]. ZnT5B has a bidirectional transport function [56]. Transporters located on the basolateral membrane of the enterocyte include ZnT1 [44], ZIP14 [102] and ZIP5 [103]. ZnT1 transports zinc ions from the enterocyte into the portal vein. Zinc ions travel in the portal blood to the liver, most likely bound to citrate, amino acids [96] and transferrin [97]. At the liver, portal blood drains through sinusoids, from where some zinc is taken up by hepatocytes. The rest of the zinc joins the systemic circulation from where it can reach distant tissues such as the brain, muscle, and bone via their respective ZIP transporters. Figure created with BioRender.com

Zinc absorption/uptake primarily occurs in the proximal part of the small intestine, in the distal duodenum and proximal jejunum [39]. Zn^2+^ ion entry into the enterocyte is mediated via ZIP4 and/or ZnT5B in the duodenum and jejunum (Fig. 1). Another transmembrane ion transporter potentially involved in zinc uptake into the enterocyte is Divalent Metal Transporter-1 (DMT-1) [29]. Regardless of the transporter used, once Zn^+2^ ions are in the enterocyte, ZnT1 transports zinc from the enterocyte into the portal blood [45]. From here, these ions travel through the hepatic portal vein, mostly likely bound to ligands such as amino acids and citrate [96]. A previous study in rats found that most zinc travels to the liver in portal blood bound to transferrin [97]. At the liver, the portal vein branches, and the blood drains through sinusoids [98]. Some zinc enters hepatocytes, most likely via ZIP14 [99]. After the blood passes through the sinusoids, it is directed to the central vein, then hepatic veins and eventually the systemic circulation [98]. From the systemic circulation, zinc ions are transported to various body tissues such as the in brain, muscle, and bone [100] (Fig. 1).

There is a wide consensus that, in the systemic circulation, the majority of zinc is bound to albumin, a lesser amount bound to *α*_2_-macroglobulin, and a fraction bound to amino acids. There is a debate over whether transferrin has a role to play as a zinc carrier in the systemic circulation. Some studies suggest it may play a role [28, 29], whilst others state that transferrin does not have a primary role in the distribution of zinc [104].

### Variability in data regarding the proportion of zinc bound to albumin and ***α***_2_-macroglobulin in systemic circulation

There have been various propositions regarding the proportions of these zinc carriers, particularly albumin. While some stated 80–85% of zinc is bound to albumin [105], others stated that this figure is 60% (with the remaining 30% bound to *α*_2_-macroglobulin and 10% to transferrin) [29], or 70% [38], or 80% (with the remaining 20% bound to *α*_2_-macroglobulin) [28], and yet others believe that approximately 98% zinc in the systemic circulation is bound to albumin [106].

Regardless of the exact percentage, conditions affecting albumin concentration, such as liver cirrhosis, may affect serum zinc levels [38]. For example, patients with liver cirrhosis and hepatic encephalopathy show decreased serum zinc levels [107].

Binding of Zn^+2^ ions to the different ligands could be the reason for the concentration of “free” Zn^+2^ ions in the circulation to be low (~ 0.1–1.0 nM) [96].

### Regulators of zinc bioavailability

Bioavailability is the fraction of intake that can be absorbed into the blood and can be used for physiological processes in the body. Studies suggest that the typical range of zinc absorption from the intestinal lumen into the circulation is 16–50% [29], with an average of around 33% [38].

An important factor affecting zinc bioavailability is the solubility of zinc in the intestinal lumen. Previously, it was proposed that amino acids released from protein digestion enhanced zinc absorption by increasing its solubility [108]. Recent reviews have suggested that protein levels in the diet positively correlate with zinc uptake, and the presence of animal-based protein enhances zinc absorption more than the presence of plant-based protein. However, it is still uncertain whether amino acids can enhance zinc bioavailability due to a lack of consensus among studies. Citrate is a low molecular weight ligand found in milk which is thought to have a positive effect on zinc bioavailability by forming zinc-citrate complexes, thereby enhancing zinc uptake. These complexes are found in higher concentrations in human milk than in cow’s milk. Therefore, zinc absorption from human milk is higher than cow’s milk [29]. Also, food fermentation and germination enhance zinc absorption by reducing the phytate content of food [108]. Essentially, fermentation and germination both promote endogenous phytase activity [109, 110]. Germination also facilitates de novo synthesis of phytases, enzymes which hydrolyse phytic acid [110].

On the other hand, several inhibitors decrease zinc solubility and, thereby, its bioavailability, for example, phytic acid/phytates found in food such as legumes, beans, and nuts. Phytates bind to zinc in the intestine, form insoluble complexes and thereby limit zinc absorption. Thus, zinc bioavailability from plant-based foods is lower than foods from animal sources [37]. Calcium and casein in cow’s milk may also reduce the bioavailability of zinc [25].

In addition, some medications may reduce serum zinc levels. For example, long-term use of distal-tube diuretics such as chlortalidone may result in significant zinc depletion due to increased urinary zinc excretion (hyperzincuria) [111]. Angiotensin-converting enzyme inhibitors (ACEis) or angiotensin-receptor blockers (ARBs), commonly used to treat heart failure, may cause zinc deficiency [112]. Also, when consuming or prescribing oral zinc supplements for zinc deficiency, interactions of medications must be carefully monitored. For example, orally consumed zinc may decrease the absorption of many orally taken drugs, such as alendronate and risedronate, which are used to prevent and treat osteoporosis. Similarly, zinc can inhibit the absorption of many antibiotics such as ciprofloxacin and doxycycline [113].

## Zinc homeostasis at physiological level

At the physiological level, zinc homeostasis is primarily maintained by controlling zinc absorption and excretion. Of the zinc that is excreted from the body, ~ 50% is lost via faeces [38] (including zinc in sloughed epithelial cells) [28], and the rest is lost through urine, sweat, menstrual flow, semen, loss of hair and nails, and shedding of skin [29]. The zinc absorption mechanism adapts more slowly, while zinc excretion mechanisms can alter quickly [29, 38]. During zinc deficiency, absorption of zinc can be increased up to 90% [28] and faecal and urinal excretion of zinc is rapidly reduced [29]. Certain tissues, such as the bone marrow, liver, and testes, secrete zinc into the circulation as a response to zinc deficiency. Other organs such as skin, skeletal muscle, heart, and kidney conserve their zinc levels even in zinc-deficient states [114].

### Endogenous zinc secretion

The process of endogenous zinc secretion into the intestinal lumen may play a role in maintaining zinc homeostasis [115]. There are many ways of mediating zinc secretion into the intestinal lumen, for example, via biliary, pancreatic and gastroduodenal secretions and sloughing of mucosal cells [56, 114, 116]. Zinc transport from the portal circulation into the enterocyte is mediated by ZIP5 and ZIP14 on the basolateral membrane of enterocytes. ZnT5B transporter on the apical membrane of enterocytes is a bidirectional transporter that can transport enterocyte zinc ions into the intestinal lumen and vice versa, thereby mediating both, enterocyte uptake and endogenous secretion of zinc [29, 117].

In the context of zinc secretion into intestinal lumen from the exocrine secretions of the pancreas, there are several zinc transporters that participate in this process. For example, zinc ions are transported into the pancreas from the plasma via ZIP5. These ions are then transported into zymogen granules via ZnT2 and excreted into the digestive tract as pancreatic secretions. Interestingly, zinc concentration in pancreatic tissues and secretions is influenced by dietary zinc intake. Excess dietary zinc leads to upregulation of ZnT2 in acinar cells, and restriction of dietary zinc leads to reduced zinc concentration in both pancreatic tissue and secretions. Thus, zinc homeostasis is regulated by adjusting zinc excretion through the entero-pancreatic axis.

Regardless of the pathway, some zinc in the lumen is reabsorbed into the circulation through uptake via enterocytes. Thus, the balance between the absorption of dietary zinc, and the excretion and reabsorption of endogenous zinc collectively maintain zinc levels in the body [29].

## Zinc homeostasis at the cellular level

Zinc concentration at the physiological level is determined by zinc regulation at the cellular level, which is determined by zinc transporters (Tables 2 and 3). Zinc transporters are regulated through various mechanisms, including activation of transcription, stabilisation of mRNA, modification of protein, trafficking to specific organelles, and transporter degradation. Regulatory stimuli include zinc, cytokines, hormones, endoplasmic reticulum stress, oxidative stress, and hypoxia [28].

### Effect of high and low zinc on ZnTs: how zinc regulates ZnT expression

ZnT transporters are differentially regulated by zinc levels but with some similarities in mechanisms. Table 2 presents a detailed view of ZnTs, their cellular and tissue distribution, and the stimuli and mechanisms involved in the upregulation or downregulation of the ZnTs. Essentially, high zinc levels increased ZnT1 and ZnT2 mRNA expressions [44] but decreased ZnT10 mRNA levels [68]. Interestingly, ZnT5 expression is unique and complex because high or low zinc levels have been shown to increase or decrease its expression [43]. This could be due to the B variant of ZnT5, which has a bidirectional functionality in zinc transport [29].

### Effect of high and low zinc on ZIPs: how zinc regulates ZIP expression

Table 3 details the ZIPs, and their cellular and tissue distribution, along with the stimuli and mechanisms involved in their upregulation or downregulation.

Most ZIP transporters were confirmed to increase their expression in response to low zinc levels including ZIP2 [74], ZIP3 [70, 76], ZIP4 [44], ZIP10 [86], and ZIP14 [92], while only ZIP5 expression was found to decrease [71]. There is some uncertainty regarding ZIP transporter regulation in response to high zinc levels. For example, ZIP7 levels inversely correlated with cellular zinc levels in CLN6 neurons [81] implying that higher cellular zinc would lead to lower ZIP7 levels. However, a causal link is yet to be confirmed because this finding could be confounded by the presence of CLN6 disease. In another example, dietary zinc restriction led to decreased ZIP11 expression in the mouse stomach. However, upon dietary zinc repletion, ZIP11 expression levels were not restored. This suggests that ZIP11 may be unaffected by zinc excess and possibly downregulated by zinc deficiency [89]. This unresponsiveness to zinc (in the form of dietary zinc repletion) [89] is unexpected, given the presence of multiple metal-response elements (MREs) upstream of the first exon of the ZIP11 gene [44].

### Other regulators of ZnTs and ZIPs

Zinc transporters respond to various stimuli other than zinc (Tables 2 and 3). For example, in the immune system, T-cell stimulation by phytohaemagglutinin decreases the mRNA expressions of ZnT1, ZnT4, ZnT6 and ZnT7 [47]. These observations reiterate the importance of zinc in modulating the immune response. Moreover, in dendritic cells, lipopolysaccharide stimulation during toll-like receptor signalling increased the mRNA transcripts of ZnT1, ZnT4 and ZnT6 but decreased those of ZIP6 and ZIP10 [46].

Cytokines (namely IL-6) are known to increase ZIP14 levels [59] but decrease ZnT10 levels [44]. Hormones such as glucocorticoid and prolactin can increase ZnT2 levels [44], while thyroid hormone can increase ZIP10 levels [88]. In addition, glucose was found to increase ZIP14 levels [94].

Metals other than zinc, such as manganese [67] and iron [91, 95], can also regulate zinc transporters, which reflects their role in assisting general metal homeostasis. Interestingly, high manganese intake upregulated liver ZIP14 expression in male and female mice but upregulated ZIP14 expression in the small intestine of only male mice. ZnT10 expression was upregulated in the same regions but only in male mice [67]. These observations indicate that there might be sex-based differences in the regulation of zinc transporters.

### Metallothioneins (MTs): at the interface of physiological and cellular zinc regulation

MTs are a family of proteins, ubiquitously expressed (in most cells and tissues), which have a high affinity for d10 electron configuration metals, including zinc and copper [118]. MT1 and MT2 are the main isoforms expressed in most adult mammalian tissues. MT3 has been identified in the brain, kidney, breast, pancreas, intestine, and bladder. MT4 has been reported in stratified squamous epithelium around the body and plays an important role in cell differentiation [119].

MTs are thought to play a key role in the systemic regulation of trace elements, including that of zinc [35, 118]. To execute this function, MTs within the enterocyte exhibit their regulatory effect at the absorption stage. Here, MTs can bind to zinc ions within the enterocyte cytoplasm and thereby reduce the availability of free intracellular zinc. Also, when zinc is needed by the cell, MTs can unbind zinc ions and make zinc available. So, if enterocyte zinc concentration is high, then MTs can bind to intracellular zinc and reduce free zinc ions [120, 121]. Consequently, this would reduce the amount of zinc exported into the portal blood, which, in turn, would reduce the amount of zinc distributed around the body. MTs are also thought to mediate zinc trafficking within the cell and zinc transfer to zinc transporters. Thus, through their zinc buffering and muffling properties, MTs help in maintaining zinc homeostasis [29].

## Acquired zinc deficiency: diagnosis and treatment

Acquired zinc deficiency could be due to insufficient intake (seen in anorexia nervosa), increased loss (seen in chronic diarrhoea or burns patients), increased requirement (seen in pregnant and breastfeeding individuals) or malabsorption (seen in Crohn’s disease [1] and coeliac disease [122]). It shows clinical features like diarrhoea, frequent infections, and skin lesions. However, these patients usually present the symptoms later in life alongside the aforementioned factors [123]. Due to overlap of symptoms, other differentials that should be considered whilst diagnosing zinc deficiency include: depression, hypothyroidism, vitamin (A, B12 and D) deficiencies, and iron deficiency [1].

Mild zinc deficiency can manifest clinically with serum values ranging from 40 to 60 µg/dL [124]. It has been suggested that acquired zinc deficiency can be diagnosed by a simple blood test showing fasting serum zinc < 70 µg /dL. Furthermore, since low albumin levels can cause low zinc levels, serum albumin levels should also be measured [125].

Some suggest that plasma zinc as a biomarker is non-specific, and it is difficult to develop a single biomarker of zinc status due to zinc’s diverse functions [25]. However, taking a fasting sample in the morning, separating plasma or serum from cells within 45 min and using zinc-free vacuum tubes can improve accuracy [1]. In general, urinary zinc levels are not a useful diagnostic parameter for zinc deficiency, whereas hair zinc levels are useful only in the context of chronic deficiency [1, 126]. In addition to laboratory investigations, the clinical aspects comprising patient risk factors, geographical prevalence, and age of presentation, alongside physical examination and an appropriate history-taking, can help to establish the diagnosis [1].

Oral zinc supplementation, such as zinc gluconate for either short-term or long-term depending on the underlying aetiology, is usually used to cure the acquired deficiency [1]. Interestingly, zinc supplements can be formulated as zinc oxide or as salts with acetate, gluconate and sulphate [2]. A clinical trial reported that zinc oxide administered without food is less well absorbed than other zinc formulations as it is more insoluble [127]. Zinc citrate has a relatively higher zinc content, yet this is countered by the finding that zinc absorption in the form of citrate does not differ from that of zinc gluconate. However, the affordability of zinc citrate may make this an attractive alternative to zinc gluconate [127]. A potential complication of zinc deficiency treatment is overcorrection with zinc supplementation since this can cause acute zinc toxicity [1]

Other clinical conditions that may show low zinc levels are tabulated in Table 4.

**Table 4:** Examples of clinical conditions that show low zinc levels

Condition/disease	Possible reason for low zinc level and the clinical status
Infection with HIV	Reduced absorption of zinc from foods. These patients often have diarrhoea, which causes excess zinc loss, resulting in low serum zinc [128]
Chronic kidney disease	Serum zinc levels tend to be on the lower side due to inadequate dietary intake, malabsorption and zinc removal during haemodialysis [129]
Liver diseases	Alcoholic hepatitis patients showed lower zinc levels compared to non-alcoholic liver disease patients [130]. Patients with alcoholic liver disease often have poor diets low in zinc whilst in cirrhosis, absorption may be impaired and there usually is increased urinary zinc excretion [131]
Polycystic ovarian syndrome that increase oestrogen levels [132]	High levels of oestrogen can decrease plasma zinc levels and increase zinc in the liver [130]
Sickle cell disease or beta thalassaemia	These patients require frequent blood transfusions, which lead to iron loading. The latter is tackled via iron chelation, but this could lead to zinc deficiency, a common complication of sickle cell treatment [133]

## Inherited zinc deficiency acrodermatitis enteropathica: diagnosis and treatment

**Fig. 2:**
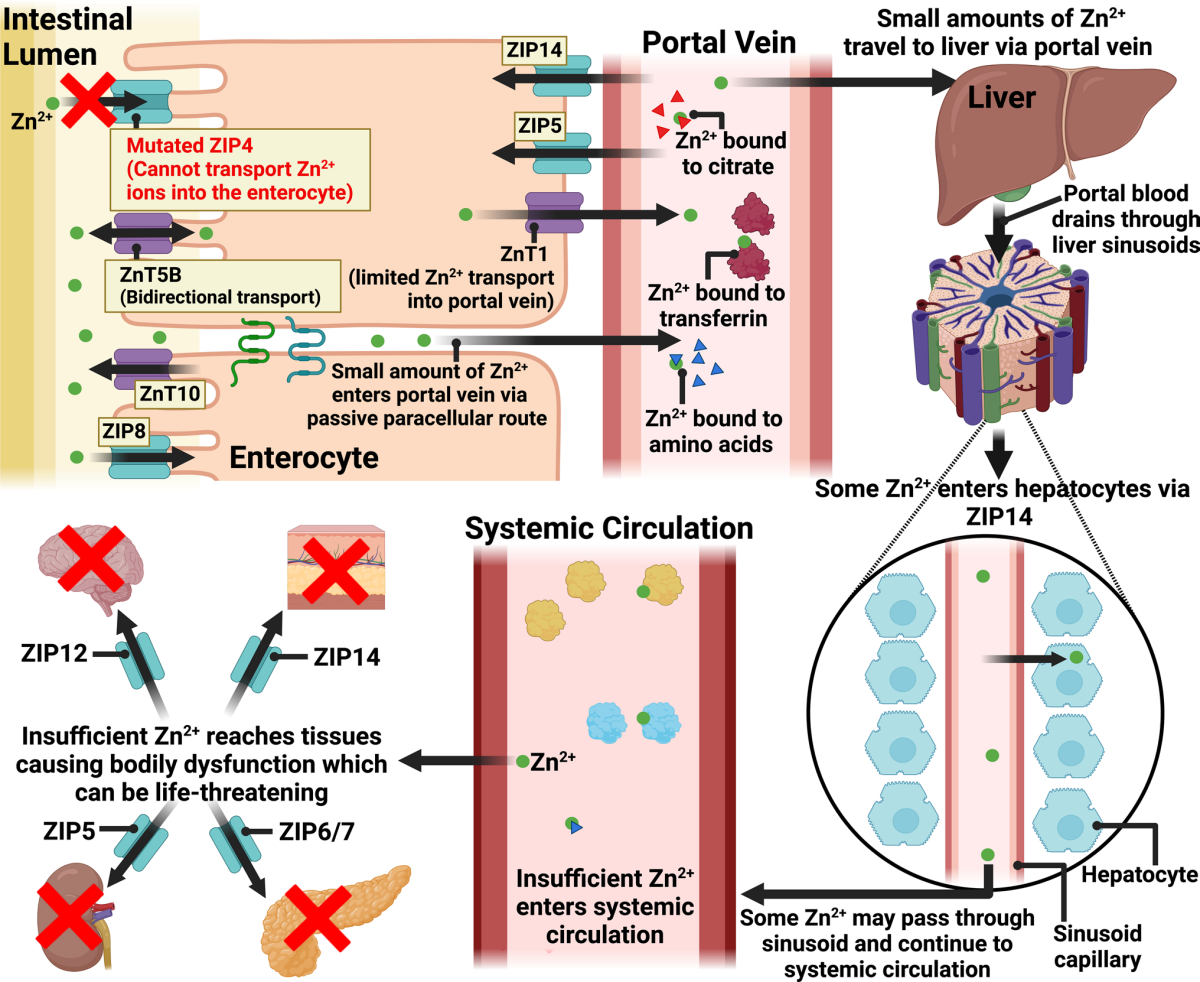
Mechanisms/events underlying zinc deficiency due to mutation in ZIP4 (Acrodermatitis enteropathica). In acrodermatitis enteropathica, there is a mutation in the *SLC39A4* gene which encodes the ZIP4 protein. Dysfunctionality in ZIP4 transporter causes limited zinc uptake by the enterocyte, and therefore, insufficient zinc transported into the portal vein via ZnT1. Insufficient zinc ions enter the liver and the systemic circulation, leading to less zinc reaching other tissues. The result is zinc deficiency, which can be life-threatening, if not treated promptly. Figure created with BioRender.com

Many inherited defects of zinc deficiencies are known. Most cases are associated with mutations in the *SLC39A4* gene on chromosome 8. This gene encodes the zinc transporter ZIP4 [134–136]. The pathological condition is referred to as acrodermatitis enteropathica, a rare autosomal recessive condition with the incidence of roughly 1 in 500,000 births [1, 137]. It affects males and females equally [137]. Because ZIP4 mediates the transport of zinc ions from the intestinal lumen into the enterocyte, a mutation in the gene encoding ZIP4 does not allow zinc ions to be transported into the enterocyte through this transporter. Consequently, insufficient zinc ions reach the systemic circulation or distant tissues (Fig. 2) [29]. Although a small amount of zinc may be taken up via the passive paracellular route [138], the result is zinc deficiency.

Notably, while ZIP4 has two zinc-binding sites and thereby can show increased efficiency in capturing and delivering zinc to the enterocytes, how ZnT5B (another zinc importer on the enterocyte) transports zinc ions into the enterocyte is not known [139]. It is conceivable that ZnT5B may have a lower affinity to zinc ions than ZIP4, and therefore, although it can allow the entry of zinc ions into the enterocyte, it cannot compensate for ZIP4 dysfunction. Left untreated, acrodermatitis enteropathica is fatal within the first few years of life [125]

Acrodermatitis enteropathica patients usually manifest symptoms early in life [1] in the phase of weaning from breastfeeding [140]. Symptoms include a triad of alopecia, diarrhoea, and dermatitis [141]. Patients may also show growth impairment, psoriasiform lesions (well-defined scaly plaques most often found on the elbows) and frequent infections [1]. Alongside the consideration of clinical symptoms, serum zinc level < 70 µg/dL in fasting and low serum alkaline phosphatase may be suggestive of acrodermatitis enteropathica [125]. Note that alkaline phosphatase is a zinc-dependent enzyme [142]. Molecular genetic testing can identify *SLC39A4* mutation and confirm acrodermatitis enteropathica.

Treatment involves zinc supplementation, but the formulation of zinc depends on the route of administration. For example, zinc gluconate and sulphate [1] are commonly used orally, while zinc chloride is preferred parenterally [125]. Treatment is lifelong with patient compliance being crucial [40].

Another genetic cause of acrodermatitis enteropathica is due to a mutation in the *SLC30A2* gene of the breastfeeding mother. This gene encodes for ZnT2, a zinc transporter expressed in the mammary glands [143]. In secreting mammary epithelial cells, ZnT2 imports zinc into vesicles, mediating zinc secretion into the breast milk. A mutation in this gene results in decreased zinc secretion into the breast milk. This can lead to severe zinc deficiency in exclusively breastfed infants [44]. This can be treated by supplementation of zinc at 5 mg per day whilst breastfeeding. After weaning, no further action is needed [143]. Most paediatric patients with acrodermatitis enteropathica do not present with the classic triad of periorificial and acral dermatitis, diarrhoea, and alopecia. Less than one-third of paediatric patients present in this way. Common presentations in children include recurrent infections, irritability, behavioural changes, neurological disturbances, and failure to thrive [140].

ZIP8 mutations result in cortical atrophy and, consequently, intellectual disability in the affected patient. A mutation in the *SLC39A14* gene (encoding ZIP14) can lead to parkinsonism-dystonia in children [144], whilst a mutated ZIP13 protein is responsible for the spondylocheirodysplastic form of Ehlers-Danlos syndrome [145]. In mice, ZIP7 knockout was lethal, whilst a morpholino knockdown of ZIP7 caused neurodevelopmental issues in zebrafish [144].

## Zinc toxicity: diagnosis and treatment

To our knowledge, there have been no reports on zinc overload/toxicity due to mutations in zinc transporters. The reported cases of zinc toxicity are due to acquired causes rather than inherited ones. Causes include pesticide exposure and exposure to compounds used to make paints, rubber and dyes [40].

The tolerable upper intake level of zinc, according to the US Institute of Medicine, is as follows: 4 mg in youngest infants, 12 mg in children 4–8 years old, 34 mg in adolescents (14–18 years), and 40 mg for persons aged 19 or older [146]. Acute and chronic zinc toxicities are defined as zinc intake of more than 200 mg/day and 50–150 mg/day, respectively [40]. Acute zinc toxicity is likely due to excessive zinc supplementation as opposed to excessive dietary zinc intake. Longer-term causes of zinc toxicity include occupational exposure to zinc [147] and iatrogenic causes such as overprescribing of zinc-containing medication, zinc present in dental fixtures (though modern preparations in the UK and US are now zinc-free) and overconsumption of over-the-counter zinc supplements [148].

At zinc doses higher than 50 mg/day, symptoms such as nausea, diarrhoea and abdominal discomfort may occur, whilst doses higher than 150 mg/day can adversely affect the body’s lipid profile and immune system. On the other hand, it has been suggested that symptoms of zinc toxicity may not manifest until intake exceeds 1–2 g [30]. The most common cause of zinc excess is taking too many zinc supplements [149]. Chronic zinc toxicity can lead to disturbances in copper metabolism causing low copper status, which affects iron distribution and causes anaemia, red blood cell microcytosis, neutropenia and reduced immune function [150, 151].

Zinc toxicity presents in different ways depending on the mode of zinc overload. For example, acute dietary ingestion presents as nausea, vomiting, diarrhoea, and muscle cramps. If toxicity is caused by inhalation of fumes, it presents with flu-like symptoms such as cough, fever, and chills. Chronic ingestion slowly leads to a syndrome of neuropathy, anaemia, fatigue, and spasticity. The 2017 Annual Report of the American Association of Poison Control Centres’ (AAPCC) National Poison Data System (NPDS) reported 1236 cases of exposure to zinc compounds, most of which were unintentional exposures in children less than five years of age. There were no deaths or major adverse health events as a consequence of this though [30].

An investigation to diagnose zinc poisoning includes several aspects like a thorough history to gain an understanding of the mode of overload, levels of serum zinc, copper and ceruloplasmin, liver function tests, platelet count, and chest X-ray. Treatment for acute ingestion involves anti-emetics, fluids and proton pump inhibitors [30]. Treatment for metal fume inhalation focuses on oral rehydration, anti-pyretics and supplemental oxygen with bronchodilators [152]. In chronic zinc toxicity, first the identification and then the removal of the source of zinc is essential. This can be followed by treatment with copper sulphate [30]. This treatment works because copper competes with zinc for absorption, so exogenous copper intake reduces zinc absorption [1]. Very severe cases may require zinc chelation with agents such as diethylenetriamine pentaacetate (DTPA) [30].

### Zinc-induced copper deficiency (ZICD)

An important complication of chronic zinc toxicity is zinc-induced copper deficiency (ZICD). Excess zinc levels in the small intestine stimulate increased expression of MTs in the enterocytes. Since copper has a greater affinity for MTs compared to zinc, copper outcompetes zinc for MT binding sites, and consequently, the copper bound to MT is excreted via sloughing of enterocytes. This results in decreased absorption of copper and, therefore, copper deficiency [153]. The co-existence of hyperzincaemia (high zinc in serum) and hypocupraemia (low copper in serum) is suggestive of ZICD [154]. Measurements of urinary zinc can be useful in the diagnostics of ZICD because urinary zinc levels are usually high in this condition [155].

ZICD tends to develop slowly over many months or years, although this apparent slow onset may be due to delayed diagnosis [155]. Early manifestation includes neutropenia, leukopenia and anaemia [154]. This anaemia is likely due to the disruption of copper’s physiological role in the differentiation of haematopoietic stem cells as well as intestinal iron absorption [156].

Under physiological conditions, copper plays a vital role in the synthesis and stabilisation of myelin, and in several enzymatic pathways required for the functioning of the nervous system. Therefore, if ZICD is left undiagnosed, it can lead to severe and permanent neurological complications including gait disturbances, paraesthesia and myelopathy [156].

One study highlighted that the over-prescription of zinc was a significant cause of ZICD. This shows that zinc can have potentially serious side effects, and it is not a harmless agent that can be prescribed without a strong justification [155]. Removal of the source of excess zinc along with oral copper gluconate treatment is often sufficient to revert anaemia, neutropenia and leukopenia seen in ZICD. Neurological deficits may also improve with this treatment; however, many never fully recover and will be left with permanent neurological deficits [156].

## Summary

Trace elements play an important role in human health and disease. For example, the role of iron in various diseases, including liver fibrosis, alcohol-related liver disease, and COVID-19, has been reviewed [157–160].

Zinc is an essential micronutrient which cannot be stored in significant amounts, so regular dietary intake is vital. There does not seem to be a clear consensus on the recommended zinc intake, where the recommendation ranges from 7.4 to 15 mg/day. Citrate and food processing such as fermentation and germination, can enhance zinc uptake. It is unclear whether amino acids enhance zinc uptake. Phytic acid, found in cereals, legumes, and nuts, is known to decrease zinc bioavailability.

Zinc absorption occurs primarily in the proximal small intestine, where ZIP4 mediates zinc entry into enterocytes. ZnT5B and DMT-1 are also thought to play a role in this process. ZnT1 transports zinc from the enterocyte into the portal vein, via which zinc travels to the liver. Here, some zinc may enter the hepatocytes via ZIP14, and the rest may eventually drain into the systemic circulation for distribution to various bodily tissues. In the circulation, most zinc is bound to albumin, and the majority in the body can be found in skeletal muscle and bone.

Zinc deficiency can be inherited or acquired. The acquired form is due to insufficient intake, malabsorption, increased requirement, or excessive loss of zinc. Inherited zinc deficiency is mostly associated with mutations in the *SLC39A4* gene (which encodes for ZIP4), resulting in a triad of alopecia, diarrhoea and dermatitis in a condition known as acrodermatitis enteropathica. In both inherited and acquired deficiencies, oral zinc supplementation is the mainstay of treatment with an excellent prognosis.

Zinc toxicity is only known to be acquired, not inherited, and may be acute or chronic. Symptoms include nausea, diarrhoea and abdominal discomfort but may vary depending on the mode of overload. Treatment involves the chelation of excess zinc using drugs.

## Recommendations for future work

This review helped us identify the knowledge gaps in the literature on zinc. For example, there is no clear consensus on the proportion of zinc that is bound to albumin in the systemic circulation. Albumin levels decrease in several conditions, including liver cirrhosis, and so, knowledge of the proportion of zinc bound to albumin may inform alternative or supplementary treatments for those with albumin-depletion-induced zinc deficiency. In such patients, only oral zinc supplementation may not be enough to resolve the deficiency because there is an insufficient level of the zinc carrier albumin to distribute zinc around the body. Should albumin be the predominant zinc carrier in the circulation, albumin supplementation may play an important role in treating zinc deficiency. There are some other uncertainties in the context of zinc carriers. For example, there are contrasting suggestions on whether transferrin has a role to play as a zinc carrier in the systemic circulation or not. Thus, the knowledge on zinc carriers will aid in our understanding of the pathogenesis of zinc-related conditions.

Another knowledge gap exists in the mechanisms by which the levels of certain zinc transporters increase or decrease in cells in response to a stimulus, as reflected in Tables 2 and 3. Dedicated studies are required to elucidate these mechanisms as this might help devise ways of altering zinc levels within cells in a tissue-specific manner and thereby help ameliorate a zinc-related diseased state. Also, while our knowledge so far indicates that zinc toxicity is not caused by mutations in zinc transporters, examining genetics as a predisposing factor in the development of zinc toxicity might be helpful, as it has been in the case of zinc deficiency. Given the multi-faceted physiological role of zinc, such studies would improve the diagnostics and therapeutics of a range of conditions, positively impacting the health of the general population as well as of those with zinc-related diseases.
